# Interventions for preventing or treating malnutrition in homeless problem-drinkers: a systematic review

**DOI:** 10.1186/s12939-018-0722-3

**Published:** 2018-01-16

**Authors:** Sharea Ijaz, Helen Thorley, Katie Porter, Clare Fleming, Tim Jones, Joanna Kesten, Loubaba Mamluk, Alison Richards, Elsa M. R. Marques, Jelena Savović

**Affiliations:** 1 The National Institute for Health Research Collaboration for Leadership in Applied Health Research and Care West (NIHR CLAHRC West) at University Hospitals Bristol NHS Foundation Trust, 9th Floor Whitefriars, Lewins Mead, Bristol, BS1 2NT UK; 2 Bristol Medical School, Population Health Sciences, University of Bristol, Bristol, UK; 3 City Hall, Bristol, BS1 5TR UK; 4 Compass Health, The Compass Centre, 1 Jamaica Street, Bristol, BS2 8JP UK; 5 The National Institute for Health Research Health Protection Research Unit in Evaluation of Interventions, London, UK; 6 Bristol Medical School, Musculoskeletal Research Unit, Translational Health Sciences, University of Bristol, Bristol, UK

**Keywords:** Homeless, Alcoholism, Alcohol, Dependence, Problem-drinking, Malnutrition, Nutrition, Micronutrients, Thiamine, Systematic review, Supplements

## Abstract

**Background:**

Excessive drinking leads to poor absorption of nutrients and homeless problem-drinkers often have nutritionally inadequate diets. Depletion of nutrients such as vitamin B1 can lead to cognitive impairment, which can hinder efforts to reduce drinking or engage with services. This review aimed to assess effectiveness of interventions designed to prevent or treat malnutrition in homeless problem-drinkers.

**Methods:**

We systematically searched nine electronic databases and 13 grey literature sources for studies evaluating interventions to improve nutrition in homeless populations, without regional or language restrictions. Screening for inclusion was done in duplicate. One reviewer extracted data and assessed risk of bias, and another checked the extractions. Primary outcomes were nutrition status/deficiency, liver damage, and cognitive function. Secondary outcomes included abstinence, comorbidities, resource use, acceptability and engagement with intervention. Results were synthesised narratively.

**Results:**

We included 25 studies (2 Randomised Controlled Trials; 15 uncontrolled before and after; 7 surveys; 1 case-control). Nine studies evaluated educational and support interventions, five food provision, and three supplement provision. Eight studies evaluated a combination of these interventions. No two interventions were the same, and all studies were at high risk of bias. Nutritional status (intake/ deficiency) were reported in 11 studies and liver function in one.

Fruit and vegetable intake improved with some education and support interventions (*n* = 4 studies) but not others (*n* = 2). Vitamin supplements appeared to improve vitamin deficiency levels in the blood (*n* = 2). Free or subsidised meals (*n* = 4) and food packs (*n* = 1) did not always fulfil dietary needs, but were usually considered acceptable by users. Some multicomponent interventions improved nutrition (*n* = 3) but acceptability varied (*n* = 3). No study reported cost effectiveness.

**Conclusions:**

The evidence for any one intervention for improving malnutrition in homeless problem-drinkers was based on single studies at high risk of bias. Various food and supplement provision interventions appear effective in changing nutritional status in single studies. Educational and multicomponent interventions show improved nutritional behaviour in some studies but not others. Further better quality evidence is required before these interventions can be recommended for implementation. Any future studies should seek the end user input in their design and conduct.

**Trial registration:**

Registered with PROSPERO: CRD42015024247.

## Background

Problem-drinking is common among homeless people [1, 2]. Homeless people are not just those sleeping rough on the street, but also include the ‘hidden homeless’ staying with friends or family, in hostels or bed and breakfasts, or in other vulnerable housing situations [3]. Problem-drinking is a variably defined term and the concept can refer to alcohol abuse without physical dependence [4, 5] or beyond ‘safe’ social drinking [6], or drinking above recommended levels and having problems in life as a result [7, 8].

Problem-drinkers tend to obtain a large proportion of their energy intake from alcohol [9, 10]. However, an alcohol-rich diet lacks important vitamins and minerals [11] and also reduces the absorption of nutrients by damaging the gut [12]. When left untreated, this can lead to impairment of cognitive, liver, and immune function [13–16]. Homeless people are at risk of being malnourished due to several factors, such as low income, limited knowledge and choice of food, and lack of cooking and storage facilities [17, 18]. The combined effect of homelessness and problem-drinking increases the risk of malnutrition [19].

Malnutrition costs around £19.6 billion per year to the public taxpayer in England and accounts for 33% of the hospital inpatient costs [20]. In December 2016 the homelessness charity Shelter said a lower-end estimate of the number of homeless in England was 250,000 from official datasets [21]. The number of malnourished individuals in sheltered housing in England is estimated to be 22% higher than that in hospital inpatients [20]. An approach to countering malnutrition is improving the nutritional quality of the food available to the population at risk. This could be achieved by educating people about healthy diet, distribution of nutritious meals or supplements, or advising the providers of food and healthcare how to tackle nutritional deficiencies.

Systematic reviews on interventions that either tackle homelessness or substance abuse in the homeless have been published [22–24], but none have addressed nutrition. There are reviews addressing nutrition in housed problem-drinkers [25, 26], however, these will not necessarily be applicable to homeless problem-drinkers. Similarly, nutrition interventions that are in line with NICE guidelines [27] are considered cost effective for addressing malnutrition in the general population [20], but information on cost effective interventions for homeless drinkers is not available for decision makers.

This review aims to bring all these elements together and synthesise evidence on interventions for improving the nutritional status of homeless problem-drinkers [28].

## Methods

### Inclusion criteria

We included studies of any controlled or uncontrolled studies evaluating an intervention that aimed to improve the nutritional status, or macro- or micro-nutrient deficiencies in any problem-drinkers experiencing homelessness. We used the UK definition of homelessness in this review which includes: sleeping rough (outside); residing in temporary accommodation such as hostels, bed and breakfasts or night shelters; staying on a temporary basis with family or friends (‘sofa surfers’); currently housed people who are at risk of being evicted; and currently housed people who cannot stay because they cannot afford to stay, the home is in a very poor condition or they are subject to violence, abuse or threats in the home [29]. Problem-drinking is a commonly used term with no agreed definition. We therefore included all definitions of problem-drinking, as defined by included study authors [28].

We did not restrict inclusion of studies based on reported outcomes. The primary outcomes of interest for this review were: nutrition status or deficiencies; liver or bone marrow damage; and cognitive function. Secondary outcomes included: mortality; suicide; incidence of acute or chronic gastritis or pancreatitis; quality of life or well-being measures; and abstinence. We also collected process outcomes such as resource use, and engagement with or acceptability of interventions.

We excluded position papers; editorials; commentaries; qualitative studies; interventions solely aimed at improving the housing status of individuals or focused solely on reducing or stopping alcohol intake; institutionalised people; studies where entire communities are homeless (e.g. refugees or occupiers of slums or shanty towns). We also excluded studies on orphans or children in state care if not part of homeless families.

### Literature searches and study selection

A search of nine electronic databases and 13 grey literature sources was conducted. References of included studies were screened and authors were contacted to find any additional studies or data. The search was published in the protocol [28] and is up to date until 16th November 2016.

References identified in searches were screened in duplicate with disagreements resolved by discussion or a third reviewer. Identified relevant papers were read in full and assessed for inclusion in duplicate, with disagreements resolved by a third reviewer. Experts and homelessness charities were contacted to find unpublished studies and data.

### Data extraction

We extracted study details including aim of the study, country, participant characteristics, sample size; outcomes reported; and outcome data including treatment effect estimates, *p*-values, and confidence intervals. Data were extracted by one reviewer and checked by another. Discrepancies were resolved through discussion (with a third reviewer where necessary). Where data were unclear, we attempted to contact the authors for clarification.

### Risk of bias assessment

Risk of bias was assessed as part of data extraction. The Cochrane risk of bias tool [30] was used for the randomised controlled trials (RCTs) and the criteria listed by the Cochrane Effective Practice and Organisation of Care (EPOC) Group were used for other types of studies [31].

### Data synthesis

Meta-analysis was inappropriate because studies were heterogeneous with respect to their populations, interventions, and outcomes. Instead we carried out a narrative synthesis.

We analysed studies by intervention type: education/information and support; food provision; supplement or fortification; multicomponent interventions. The findings were summarized in tables for the main outcomes.

## Results

### Description of included studies

#### Results of the search

**Fig. 1:**
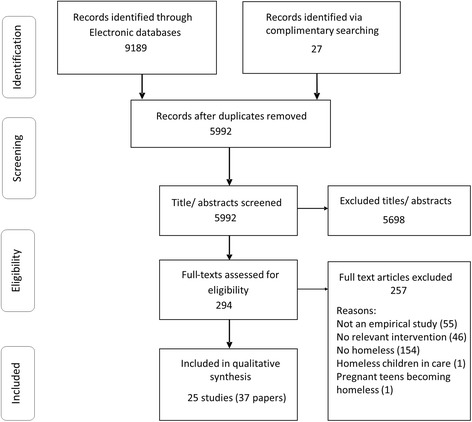
PRISMA flow diagram of the review process

Electronic searches resulted in 9189 citations. Twenty seven other references were identified in complimentary searches (contacting authors/ organisations, reference screening). In addition, we identified two further studies through contacts with subject experts [32, 33] but the authors did not provide sufficient information to assess eligibility to date. We included 25 studies reported in 37 articles / reports and excluded 257 papers (web appendix). The selection process and reasons for exclusion are shown in the PRISMA flowchart (Fig. 1).

#### Included studies

Of the 25 studies included, the majority (*n* = 12) were from the USA [34–45], and the rest were geographically diverse. Table 1 provides the characteristics of all included studies. Two studies [34, 41] were RCTs, both from the USA; fifteen were uncontrolled before and after (UBA) studies [35–37, 39, 40, 43, 44, 46–53]; seven were surveys [38, 42, 45, 54–57]; and one was a case-control study using a historical control sample [58].

**Table 1:** Characteristics of included studies

Study	Location	Study design	Participants					Intervention	Comparison	Primary outcomes reported^a^
			N	Inclusion criteria	Recruitment	Homeless type (shelter/ rough sleeper)	Heavy drinkers			
Educational information or support interventions										
Rusness 1993	USA	UBA	7	Homeless women at a shelter	Shelter	Shelter	NR	Biweekly classes focused on nutrition information, shopping and cooking skills	No control group	Nutritional status
Hinton 2001	UK	UBA	18	Residents at the homeless shelter	A homeless shelter	Shelter dwellers	NR	a session on food hygiene and nutrition, a cooking competition	No control group	No^b^
Derrickson 2003	USA	RCT	210	Households at risk of homelessness who requested assistance between January to August 2001	NR, likely from the database of the Salvation Army Family Services Office	At risk households	NR	3-h nutrition workshop	1-h food safety workshop.	Nutritional status
Heslin 2003	USA	Comparative survey	974	Homeless women of reproductive age in Los Angeles County shelters and meal programs.	Shelters and meal programs	NR, likely all types	NR	case manager assigned to optimise uptake of WIC	Homeless women in WIC without case manager	No
Helfrich 2006	USA	UBA	32	Self-identify a life skill need, be willing to engage in sessions each week, able to give informed consent and understand English	Shelters/ emergency shelters, transitional/ emergency housing program	Shelter	NR	Life-skills workshops & individual sessions	No control group	No
Johnson 2009	USA	UBA	50	Long-term residents in the shelter (2 to 6 months), have at least one child residing with her in the shelter, and is enrolled in the shelter’s life skills program	Two homeless shelters	Shelter	NR	Nutrition education classes	No control group	Nutritional status
Bonevski 2012	Australia	UBA	6	> 18 years, English speaking, receiving accommodation support from the participating homeless centre	A non-government homelessness outreach centre	NR, likely shelter	58%	Telephone personal counselling on health	No control group	Nutritional status
Rustad 2013	USA	UBA	118	English-speaking, low-income women living in the Minneapolis/ St Paul area	Soup kitchens, grocery stores, Laundromats, food shelves, and homeless shelters	NR, likely shelter or in transition	NR	3 nutrition and health education sessions	No control group	Nutritional status
Barbour 2016	Australia	UBA	5	Young person engaged with case management services in the community agency, with an interest in eating healthier and improving their cooking skills	Agencies helping homeless youth	Crisis accommodation, sleeping rough and couch-surfing	NR	Food literacy programme, participants engaged in a 3-h group interactive session over 8 weeks	Daily recommended values DRVs for males of age 19–50 years	Nutritional status
Fortification / Supplement Interventions										
Darnton-Hill 1986	Australia	Comparative Survey	106	Quasi random selection: the first three attendees of the homeless shelter/clinic; first person sitting left of the entrance plus two more at the day centre	Homeless shelter, day centre, and a clinic	NR, Likely shelter and rough sleepers	70%	Men taking oral multivitamin	Not taking vitamins	Nutritional status
Drijver 1993	Netherlands	UBA	9	Almost daily alcohol consumption for past 5 years; average use of 8 E (80 g) alcohol per day; age 20–65 years; no vitamin supplements in the past month; thiamine level < 110 nmol	Homeless houses and outpatient facilities for alcoholics	NR, likely all types	100% (all drinking > 5 years, 80 g or more /day)	Single or weekly Intramuscular injection of combined 200 mg thiamine, 100 mg pyridoxine, 1000 ng cyanocobalamin	No control group	Nutritional status
Darmon 2009	France	Repeat Survey	130	Men attending any of the 8 emergency shelters in Paris (3 night shelters and 5 food aid day centres)	Emergency shelters	NR, Likely shelter and rough sleepers	NR, likely majority	Fortified chocolate spread distribution	No control group	No
Food provision interventions										
Garden 2013	Russia	Case (historical) control	142	All homeless patients with tuberculosis referred to a St. Petersburg’s Tuberculosis dispensary	Tuberculosis dispensary	NR, likely all types	45% (registered alcoholics)	Daily food packs including canned meat, bread, butter, egg and soup with cream, juice, tea and yoghurt (2000 kcal)	Homeless treated at the tuberculosis dispensary in previous years	No
Murakami 2013	Brazil	UBA	315	Low income people (elderly, unemployed, homeless and itinerant) who have been to the restaurant ≥3 time per week	NR	NR, likely all types	NR	Low cost meals available at restaurants	No control group	Nutritional status
Villena 2013	Spain	Survey	50	Clients coming to the meal provision centre	Community kitchen	NR, likely all types	NR	Evaluating five community kitchen menus	No control group	No
Pelham-Burn 2014	UK	Survey	16	Clients coming to a meal provision centre	The lounge area / front desk of the meal provision centre	NR, likely all types	NR	Taste testing 12 lunch dishes.	No control group	No
Allen 2014	Australia	UBA	78	Rooming house residents, homeless persons and others deemed eligible for entry to the project	Café Meals project database North Yarra Community Health	All types	19% alcohol dependent	Providing clients a subsidy that entitles them to one meal per day at one of four local cafés	No control group	Nutritional status
Multicomponent interventions										
Wiecha 1993	USA	Comparative Survey	77	Homeless families without overt substance abuse or emotional problems with a child under 6 placed by the public welfare in temporary accommodation	Shelters and meal programs	Transitional homeless	NR	Kitchen facilities without food support (shelter) versus facilities & food support (shelter)	No kitchen facilities or food support (hotels)	Nutritional status
Tarasuk 1994	Canada	UBA	49	Homeless adult attenders of an inner city drop in centre	Drop in centre for homeless adults	All types	NR	Three sequential interventions: 1) weekly cooking classes; 2)making the centres’ kitchen available for use to street-living; 3)communal cooking and dining	No control group	No
Hamm 1999	USA	UBA	31	families in transition- who are temporarily living in shelters, transitional housing or with friends/family	Homeless shelters, soup kitchens, transitional housing, nurseries and day-care centres and family support centres	Transitional homeless	NR	Group nutrition education classes, health checks and food pack vouchers useable at specified stores	Non-homeless WIC participants	No
Stewart 2009	Canada	UBA	56	Homeless or in transition homeless youth	An employment programme and drop-in centres	All types	34% sought counselling for alcohol/ drugs	Weekly support groups (help with homework, course or job finding, recreational activity, meal, transport)	No control group	No
Richards 2011	USA	Comparative Survey	11,181	Homeless pregnant women with complete data in the PRAMS database	PRAMS database	All types	NR	WIC homeless women	Non WIC homeless women	No
Kadoura 2014	USA	UBA	25	Homeless families with at least one child at the shelter school. Speak English or Spanish.	Homeless shelter	Shelter	60% parents reported drug and alcohol use substance abuse	10 two-hour sessions, including physical activity, education/training, and a ‘healthy dinner’	Non concurrent national data	Nutritional status
Grazioli 2015	USA	UBA	6	Homeless drinkers, with a disability; homeless for at least 1 year or on 4 or more separate occasions in the past 3 years; aged 21–65 years	2 community-based agencies	NR, likely all types	100%	Safer-drinking strategies: treatment with extended-release naltrexone and harm-reduction counselling	No control group	Liver function
Kendzor 2016	USA	RCT	32	≥18 years of age; willing+ able to attend all visits; > 6th grade literacy level; able to walk; resident of the transitional shelter for ≤2 months.	One shelter	Shelter dweller	NR	Newsletters, fruit/veg provision & pedometers/ walking goals.	No Intervention: Paid assessment-only	Nutritional status

*Kcal* kilo calories, *N* number of participants analysed, *NR* not reported, *PRAMS* Pregnancy Risk Assessment Monitoring System project for CDC, USA, *RCT* randomised controlled trial, *UBA* uncontrolled before after study, *WIC* The Special Supplemental Nutrition Program for Women, Infants, and Children in the USA. ^a^ Primary outcomes of the review that were reported in the study. ^b^ This means that the study did not measure or report any of the primary outcomes of this review

Studies varied widely in sample size from 5 participants [53] to 128,365 [42], with a median of 50. Most studies (*n* = 17) included less than 100 participants. The total number of participants included in this review is 131,054. Most studies (*n* = 9) did not report on type of homelessness. Six included shelter dwelling participants only [37, 39–41, 43, 50] while no study included just rough sleeping participants. Problem-drinking was not always defined. Only two studies provided a clear definition of problem-drinking as more than 80 g alcohol intake per session or per day [46, 56], while other studies used terms such as ‘alcoholics’, ‘alcohol dependency’, ‘overt alcohol problem’, ‘drinking at risky levels’, or ‘seeking counselling for alcohol’. Proportion of problem-drinkers in the study population also varied (median 45%, IQR 26.5% – 64%) across studies that reported this information.

Interventions assessed in included studies were grouped into four broad categories based on the different approaches to addressing malnutrition:

1. Education, information or support
2. Supplements (including vitamin injections or tablets, or fortified food products)
3. Food provision (including hot meals or food rations)
4. Multicomponent interventions, where studies combined more than one of the above approaches

Educational, information, and support interventions were the most common (*n* = 9) [34, 37–39, 43, 44, 50, 53]. These included motivational interviewing with nutrition information over the phone [50], a one-off lecture [48], interactive group workshops [34, 37, 43], and full curriculums on nutrition and diet [39, 44, 53]. Three studies assessed the effect of supplements and fortification. These included oral or injectable multivitamin supplements [46, 54] and a vitamin-fortified chocolate paste [56]. Five studies tested food provision interventions [51, 52, 55, 57, 58] ranging from prepared meals provided within organisations [55, 57] and access to prepared meals at specified cafes [51, 52] to daily food packages given to clients at a tuberculosis clinic [58]. Multicomponent interventions were assessed in eight studies [35, 36, 40–42, 45, 47, 48]. The combination of interventions included nutrition counselling on harm reduction (e.g. to eat before drinking) with naltrexone detoxing [35], provision of education and kitchen facilities [47], provision of food along with kitchen facilities [45], education sessions with food and physical activity [40, 41], support sessions, recreational activities along with transport tickets, and free meals [49], Women, Infants and Children (WIC) programme [42], and addition of group nutrition education, health checks, and food pack vouchers to the WIC programme [36].

#### Risk of bias in included studies

**Fig. 2:**
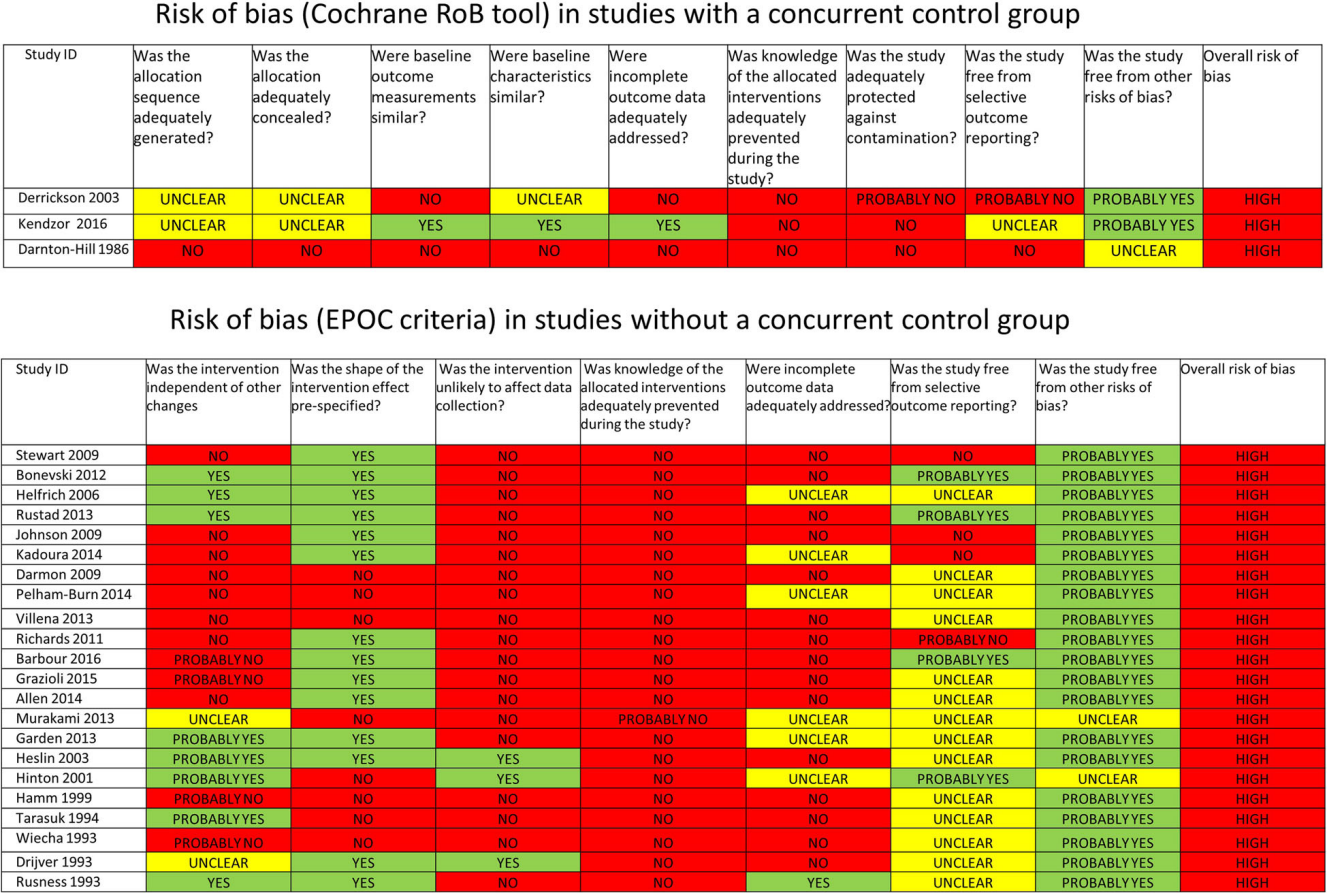
Risk of bias in included studies

All studies were considered to be at a high risk of bias (Fig. 2). In the two RCTs the sequence generation and concealment of allocation were both rated ‘unclear’. None of the studies reported attempts to adequately prevent knowledge of allocation to intervention (blinding) either for participants, providers or outcome assessors. Only two [41, 43] out of the 25 studies adequately addressed incomplete outcome data in their analyses. The majority of the studies (*n* = 19) were also judged to be at high risk for the knowledge of allocated intervention to have affected data collection (based on Cochrane EPOC criteria) [31].

### Findings

#### Primary outcomes

##### Nutritional status

Nutritional status measures were reported in 13 studies [34, 39–41, 43–46, 50–54]. Of these, three studies reported micronutrient deficiency in blood samples [43, 46, 54] and ten reported on nutritional intake [34, 39–41, 44, 45, 50–53].

##### Education, information or support interventions

Educational sessions of varying intensity and duration were assessed in four studies [34, 39, 44, 53]. One involved motivational telephone interviewing [50], one was a single session on food hygiene and nutrition along with a cooking competition [49], and one involved life skill workshops [37]. One study assigned a case manager to provide information and support to optimise the uptake of the Supplemental Nutrition Program for WIC [38]. Most of these were aimed at change in nutritional intake [34, 39, 43, 44, 50, 53]. The studies varied in design (one RCT, others uncontrolled before after studies) and outcome assessments (attempts to increase intake, frequency, or amount of intake). The effects were not consistent across studies, however, the majority indicate that education and support interventions could contribute to improved nutritional behaviour, i.e. eating healthier food (Table 2).

**Table 2:** Primary outcomes in included studies

Study	Design/ duration	N	Outcome	Findings	Direction of effect / interpretation
Education, information or support					
Rusness 1993	UBA/ 1 month	7	Number with Anaemia (%)	3 (43%)	Unclear if this is due to nutrition education classes: no pre-test values; 1 month study
			Number with Hypalbuminaemia (%)	1 (14%)	
			Eating right skill score- food frequency data (Mean change)	“One third higher than pre test scores”	Educating shelter living women in healthy eating improved nutritional intake
			Numbers of women maintaining family targeted diet behaviour (%)	6 (86%)	
Derrickson 2003 (RCT)	RCT/ 1 month	210	Mean (SD) intake of fruit servings per day in compared groups post intervention	Intervention = 6.6 (7.5) Control = 4.5 (4.8)	Nutrition workshop increased average fruit and vegetable intake
			Mean (SD) intake of vegetable servings per day in compared groups post intervention	Intervention = 8.3 (7.8) Control = 6.3 (6.2)	
Johnson 2009	UBA/ 10 months	50	Proportion who ate more fruit and vegetables compared to baseline	19%	Nutrition education classes made more people eat fruit and vegetable and yogurt, and =avoid carbohydrate
			Proportion who ate more yogurt compared to baseline	3%	
			Proportion who tried to limit carb intake compared to baseline	22%	
			Mean (SD) of fruit servings eaten daily	Pre-test = 0.83 (0.71) Post-test = 0.7 (0.65)	Nutrition education classes decreased mean fruit intake and increased carbohydrate intake
			Mean (SD) servings of bread, cereal, pasta, and rice (eaten) daily	Pre-test = 1.44 (1.16) Post-test = 1.83 (1.29)	
Bonevski 2012	UBA/ 1.5 months	6	Proportion who tried to eat more fruit N (%)	4(66%)	Intervention increased attempts to eat fruit and vegetable
			Proportion who tried to eat more vegetable N (%)	6 (100%)	
Rustad 2013	UBA/ 1.5 months	118	Mean (SD) of fruit serving intake	Pre-test = 1.3 (1.3) Post-test = 1.6 (1.4)	Nutrition and health education sessions increased fruit and vegetable intake
			Mean (SD) of vegetable serving intake	Pre-test = 1.5 (1.3) Post-test = 1.9 (1.5)	
Barbour 2016	UBA/ 6 months	5	Mean (range) fruit servings eaten/ day (compared to reference Daily recommended values)	Pre-test = 0.8 (0, 2.2) Post-test = 0.4 (0.0, 1.0)	Food literacy programme decreased mean fruit intake and mean diet quality score.
			Mean (range) vegetable servings eaten/ day (compared to reference Daily recommended values)	Pre-test = 2.7 (0.0, 11.9) Post-test = 3.6 (0.0, 12.0)	
					Intervention increased mean vegetable, iron, vitamin C, folate, calcium, and total energy intake
			Mean (range) intake of Folate (B9) mg/day	Pre-test = 256 (211, 272) Post-test = 309 (108, 551)	
			Mean (range) intake of Calcium mg/day	Pre-test = 655 (365, 998) Post-test = 771 (423, 1367)	
			Mean (range) intake of Iron mg/day	Pre-test = 9.9 (6.6, 14.4) Post-test = 10.4 (5.3, 15.9)	
			Mean (range) vitamin C intake mg/24 h	Pre-test = 67 (10, 159) Post-test = 72 (0, 143)	
			Mean (range) diet quality score (max 100)	Pre-test = 45 (38, 61) Post-test = 41 (27, 60)	
			Mean (range) daily energy intake kJ	Pre-test = 7981 (2574, 11,384) Post-test = 10,244 (6321, 15,152)	
Supplement provision					
Darnton-Hill 1986	Comparative survey/ 24 months	106	% deficient in vitamin B1	NV gp = 45 V gp = 25	Oral vitamin supplements reduced the number of people with vitamin deficiency
			% deficient in vitamin B6	NV gp = 63 V gp = 21	
			% deficient in vitamin C	NV gp = 29 V gp = 10	
			% deficient in vitamin B12	NV gp = 0 V gp = 0	
			% deficient in folate (B9)	NV gp = 80 V gp = 49	
			% deficient in iron	NV gp = 12 V gp = 15	
			% deficient in zinc	NV gp = 25 Vgp = 25	
			Mean (SD) levels of TPP%	NV gp = 15.3 (10.5) V gp = 10.5 (9.9)	Oral vitamin supplements don’t always improve group mean levels of vitamins
			Mean (SD) levels of vitamin B6 P5P%	NV gp = 57 (26.6) V gp = 36.2(31.4)	
			Mean (SD) levels of vitamin C μmol/L	NV gp = 34.9 (16.2) V gp = 72.6 (35.2)	
			Mean (SD) levels of serum Folate ng/ml	NV gp = 3.6 (4.0) V gp = 5.2 (4.0)	
			Mean (SD) levels of vitamin B 12 pmol/L	NV gp = 341 (203) V gp = 433 (223)	
Drijver 1993	UBA/ NR	9	Mean Tk activity increase (units)	Single injection: Before = 9.6; day 14 = 11.8	Multivitamin injection keeps vitamin levels up for 14 days.
				Weekly injection: Before = 10.2; day7 = 12; day21 = 11.2; day35 = 12	
			Mean TDP effect (%)	Single injection: Before = 18; day 14 = 9	
				Weekly injection: Before = 17; day7 = 3; day21 = 5; day 35 = 5	
Food provision					
Murakami 2013	UBA/ NR	315	% of Clients eating below recommended energy intake	79.0	The hot meals do not fulfil energy needs for most participants, and even though provide a high fibre diet, still contribute to higher than recommended fat and saturate intake in many participants.
			Mean (SD) 24 h Energy intake kcal	948.55 (108.75)	
			Proportion with above average fibre intake	62.9%	
			Proportion with saturated fat above the recommended levels	22%	
			Proportion with cholesterol intake above the recommended levels	41%	
Allen 2014	UBA/ 12 months	78	Proportion eating more frequently and gaining weight	Numbers not reported: “many clients eat more frequently, and experience positive weight gain”	A subsidy to have one meal per day n may increase food intake
Multicomponent interventions					
Kendzor 2016	RCT/ 1 month	32	Mean (cups) vegetable and fruit intake	Intervention = 3.56; controls =2; MD = 1.5 cups more in intervention at 4 week follow up	Newsletters, fruit/vegetables & pedometers with walking goals are able to increase fruit and vegetable intake
Wiecha 1993	Comparative survey/ 9 months	77	Mothers’ Mean (mg) Vitamin B6 intake per 1000 kcal	Kitchen facilities with or without food support (shelter group) = 0.68; no facilities or food(hotels group) = 0.55	Provision of full kitchen facilities with or without added food support can increase intake of important micronutrients but not total protein or energy intake for families
			Mothers’ Mean (mg) Vitamin C intake per 1000 kcal	Kitchen facilities with or without food support (shelter group) = 61; no facilities or food(hotels) group =41	
			Mothers’ Mean (g) protein intake per 1000 kcal	Kitchen facilities with or without food support (shelter group) = 35; no facilities or food(hotels) group =33	
			Mothers’ Mean Energy (kcal) intake per 1000 kcal	Kitchen facilities with or without food support (shelter group) = 1980; no facilities or food(hotels) group =2016	
Kadoura 2014	UBA/ 1 month	25	Mean change in frequency of fruit and vegetable intake (Cohen’s D)	0.56	Family physical activity, education/training, and a ‘healthy dinner ‘increased both amount and frequency of fruit and vegetable intake
			Mean change in amount of fruit and vegetable intake (Cohen’s D)	0.87	
Grazioli 2015	UBA/ 3 months	6	AST levels median (IQR) units	Baseline = 64.5 (34.5, 95.5), follow up = 60 (29.25, 90.5), Wilcoxon signed rank test = −0.77	Detoxification with naltrexone and harm-reduction counselling with a focus on better diet habits led to no change in liver function tests post intervention
			ALT levels median (IQR) units	Baseline = 40.5 (30.25, 51.5), follow up = 32 (21.5, 56.75), Wilcoxon signed rank test = − 0.7	

*AST*aspartate transaminase, *ALT* alanine transaminase, *B1* thiamine, *B2* riboflavin, *B3* niacin, *B5* pantothenic acid,*B6* pyridoxine, *B7* biotin, *B9* folic acid, *B12* cobalamins, *C* ascorbic acid, *g* gram, *gp* group, *kcal* kilocalories, *kJ* kilojoules, *L* litre, *MD* mean difference, *mg* milligram, *mmol* millimoles, *nmol* nanomoles, *μmol* micromoles, *N* number of participants, *NR* not reported, *NV* no vitamin, *pmol* picomoles, *P5P* pyridoxal 5 phosphate, *RCT* randomised controlled trial, *SD* standard deviation, *Tk* transketolase, *TDP* thiamine diphosphate, *TPP* thiamine pyrophosphate, *UBA* uncontrolled before and after study, *V* vitamin

##### Supplement provision interventions

The oral [54] and injectable [46] multivitamin supplements were effective in lowering blood indictors of deficiency (Table 2). However, no longer term health or disease outcomes were measured.

##### Food provision interventions

One study [51] reported on a large-scale state supported intervention providing healthy meals at designated cafes and diners. The authors reported energy intake to be below recommended levels for clients eating there daily. One study providing food subsidy for meals at a local cafe [52] reported that people ate more frequently, had weight gain and learnt food preparation skills and healthy eating habits, but quantitative data were not reported (Table 2).

##### Multicomponent interventions

Nutrition intake changes were reported in three studies [40, 41, 45]. All indicated a beneficial effect of the multicomponent interventions on healthier food intake (Table 2).

##### Liver function

Only one study [35] evaluating a multicomponent intervention (naltrexone detoxing and harm reduction counselling) reported liver function tests (mean aspartate transaminase and alanine transaminase levels) for the intervention group only, and found no difference between before and after measurements (Table 2).

#### Secondary outcomes

Change in drinking behaviour was reported in three studies [35, 40, 49]. Several studies assessed some aspects of success of implementation of their intervention/ programme, such as the acceptability of the intervention [35, 55–57], or attendance and intervention/ programme completion [36, 41, 48, 58]. Cost of interventions was reported in four studies [51, 54, 56, 58]. However, no study reported cost effectiveness analyses.

##### Drinking behaviour

Three studies, all assessing multicomponent interventions reported this outcome. Kadoura [40] reported a small non-significant decrease (Cohen’s d = 0.15; *p* = 0.15) in mean alcohol consumption compared to baseline in the intervention group of homeless families. Stewart et al. [49] reported 26% fewer participants drinking at the end compared to the mid-point assessment. Grazioli et al. [35] indicated the intervention (naltrexone + nutritional counselling) may lead to more drinking.

##### Measures of implementation success

Eight studies reported some measure of implementation success (see Web appendix for details). Poor attendance (0–22%) was seen for an intervention using education sessions with a cooking competition at a shelter [48]. Higher treatment completion rates were seen in a tuberculosis clinic for homeless with a daily food pack provision [58]. In taste testing carrot cake, beef burger, and apple crumble were liked the most in a study in the UK [57], while a study in Spain [55] found that dairy, fruits and beans were favoured, however both reported a low preference for fish. There was high acceptability for the vitamin fortified chocolate spread packets [56].

High attendance rates and perceived effectiveness were observed in a study that provided education, food provision and goal setting [41]. In contrast, low acceptability and perceived effectiveness were seen in a study of naltrexone detoxing with counselling for better eating [35]. Group nutrition education and health checks part of the WIC intervention were acceptable to homeless families. However, food voucher uptake and use was low [36] and reasons for this low uptake of vouchers were: transiency, loss of the identification documents, loss of vouchers, lack of transport, and lack of time. One third of these families reported difficulty carrying a large amount of groceries, and 23% did not think the food met their needs fully.

##### Cost and resource use

No studies reported cost effectiveness or provided enough data to assess cost effectiveness. Five studies reported cost and resource outcomes for the interventions tested [47, 51, 54, 56, 58] (Table 3). One other study [57] recorded information on costs of meals provided but did not report this data in the paper. The table shows that although some of the cost information dates as far back as the 1980s and the comparisons are indirect, vitamin tablet supplementation could be cheaper than other interventions.

**Table 3:** Cost and resource use in included studies

Study	Intervention	Outcome Measure and Findings (USD)^a^
Darnton-Hill 1986	Vitamin C, B complex, and thiamine regimen	Cost / day AUD (USD): 0.168 (0.12)
	B complex capsule	Cost / day AUD (USD): 0.085 (0.06)
	Thiamine tablet 50 mg	Cost / day AUD (USD): 0.035 (0.03)
	Vitamin C tablet 500 mg	Cost / day AUD (USD): 0.048 (0.03)
Darmon 2009	Vitamin fortified chocolate spread plus street food	Cost of one RDA diet EUR(USD): 3.64 (5.07)
	Food aid meal along with street food	Cost of one RDA diet EUR(USD): 4.78 (6.6)
	Street food alone	Cost of one RDA diet EUR(USD): 5.6 (7.7)
Garden 2013	2000 kcal day-food pack	Average cost USD: 1.3–1.5
Murakami 2013	Breakfast (400 kcal)	Cost of one meal R$ (USD): 0.5 (0.15)
	Lunch (1200 kcal)	Cost of one meal R$ (USD): 1.0 (0.31)
Tarasuk 1994	Communal cooking and dining in shelter kitchen	Staff needed to co-ordinate: 1 person

^a^USD values (In brackets) when the reported cost values were in other currencies calculated using historical exchange rates for the respective publication year’s January

Other outcomes (detailed in web appendix) reported in included studies were life skills [37, 53], infant health and health visits by mothers [42, 43, 49, 50], physical activity [41, 50], access to shelter and food [38], and social outcomes such as loneliness and enjoyment [47, 49].

## Discussion

### Summary of findings

To our knowledge, this is the first review of the evidence-base on the effectiveness and costs of interventions for malnutrition in the homeless problem-drinking population. We included 25 studies assessing four broad categories of interventions. We found that in terms of nutritional status, educational interventions may increase fruit and vegetable intake, but this was not consistent across studies. A fortnightly multivitamin injection or daily multivitamin oral tablet could prevent vitamin deficiencies. A daily multivitamin fortified chocolate spread pack was acceptable but evidence of effectiveness was not reported. Free or subsidised meals or daily food packs also appeared acceptable but did not always fulfil adult energy needs. Three multicomponent intervention studies assessed nutritional status and all showed improved nutritional intake. In terms of implementation success or acceptability, provision of food or kitchen facilities were usually well received, but education in combination with detox or a cooking competition, or food vouchers that required money and time to be redeemed were not favoured.

All studies were at high risk of bias. Many studies did not have control groups. Particular problems with interpreting data from uncontrolled studies are susceptibility to confounding (including seasonality) and regression to the mean [59]. There were two RCTs and although these were at high risk of bias too, they are more reliable than the other studies because of randomization. Both reported higher intake of fruits and vegetables in the intervention group, one providing a 3 h workshop on resource management and diet [34] and the other providing newsletters, fruits, vegetables, and pedometers along with walking goals [41]. These interventions could be implemented in shelter settings similar to those in the respective studies.

It was not possible to estimate cost effectiveness as none of the studies reported cost-effectiveness analysis. Limited data on intervention costs from four studies on food or supplement provision interventions indicate the costs of daily oral vitamin supplements to be lower than that of meals. However, this information without the benefits associated with each is of limited use when choosing between competing interventions, especially when these were not directly or concurrently compared.

There were no assessments of cognitive function in the included studies. Although this may be difficult to measure accurately, it is an important outcome which can affect people’s ability to optimally use healthcare and housing services, which may require learning and remembering new things [60]. Long term outcomes in health are also missing from the literature. These outcomes can be useful for decision makers in establishing whether the interventions provided the intended benefit. Nutritional outcomes reported across studies were ill-defined and variably measured. There are known issues in this area of research associated with use of convenient measures, transiency of the population, and the extreme variation in food intake dependent on donations or the opening days/ h of soup-kitchens or similar facilities [61, 62]. Future studies in this population should use more rigorous measures of nutritional change [62].

Nine studies reported use of incentives (cash or gifts) to increase data collection and uptake of intervention. This, along with high dropouts, suggests that effectiveness may not be entirely attributable to the intervention.

### Strengths and limitations

Given that we expected limited evidence on the question, not using language restrictions and using an extensive grey literature search was a key strength of our review. This strategy was likely the reason we were able to identify a relatively large number of studies. We supplemented this exhaustive search with rigorous methods of inclusion and appraisal of the research identified. This makes our findings reliable. Another strength of this review lies in its inclusivity. This allowed for the unrestricted inclusion of and, consequently, the exploration of the range of interventions evaluated. The results of this review can therefore inform the implementation of these interventions to improve the health of homeless problem-drinkers in similar settings. No two studies of similar design assessed the same intervention and outcome and thus an expected limitation of this review was that results of the studies could not be combined in a meta-analysis. Thus the interpretation requires caution, especially considering the limitations in study quality. We therefore analysed interventions in broad categories and avoided subgrouping of results further.

### Applicability of evidence

There are still lessons to be learnt from this limited evidence base and implications for policy and research, although caution is required when implementing this evidence.

Most interventions were set in shelters and inclusion often restricted to shelter dwellers. No study assessed an intervention for rough sleepers alone. This group often gets excluded from such research studies due to their transient location, yet they may have more complex needs [61], so their inclusion in future research would be valuable. Although some shelters do, not all will accept rough sleepers who are heavy alcohol or drug users [45, 63]. Rough sleepers with drinking or drug problems also sometimes avoid engaging with shelters [64]. Healthcare access points, such as general practices offering primary health care services to street drinkers may be a more inclusive setting to reach this subgroup [61]. Even when these are free at point of delivery, it could mean uneven reach where the most vulnerable homeless drinkers may be unaware of or unable to reach these services.

Although, education and training can raise awareness in and provide information to homeless people to make healthier food choices, it is harder for homeless people to make these healthy choices when they have no kitchen, cupboard, or fridge, and move residence frequently [39, 44]. In addition, vitamin B1 deficiency with heavy drinking over time leads to cognitive impairment [65]. Once in this state, any counselling or health promotion efforts to bring about behaviour change would be less likely to work [66]. Thiamine intake can often reverse this impairment [67]. Alcohol and/or drug support services often pursue harm reduction interventions (e.g. needle exchange) alongside interventions to reduce substance abuse [68]. These services could also consider providing nutritional interventions for preventing or treating malnutrition in the homeless, heavy drinking population.

Our findings suggest that consideration for local taste preferences is important in meal provision services. However, only one study [58] reported developing the content of the food rations according to local food tradition. Meal provision was an acceptable intervention although evidence suggested that it may not always fulfil energy needs. Meal services require a full kitchen and catering staff to serve meals every day. In addition, there is evidence that nutritional value of these meals may be constrained by resources and prioritising a satisfying meal over nutritional value by both providers and users [57]. No included study compared different types of food and/or supplement provision interventions and this should be assessed in future for comparative benefit and resources use.

Provision of kitchen facilities in two studies appeared to be effective in improving nutritional intake. It led to a reduction in vitamin deficiencies in one study in homeless families [45]. The other study [47] that made kitchens available to street-living homeless as well as the shelter participants reported that the participants enjoyed cooking, and the use of the kitchen facilities increased rapidly. This indicates that the lack of these facilities might be a key factor in malnutrition among homeless drinkers [18]. It also suggests that experiential learning may be more effective than giving nutritional advice in this client group [44].

It seems that an educational intervention with a cooking competition proposed by the shelter staff alone had low uptake [48], but an intervention developed with service user input, involving education, kitchen facility and communal dining was popular [47]. Involving the population in research can increase participation rates and user controlled research is encouraged in public health and social care research [69]. Considering the difficulty in accessing homeless drinkers, their involvement in a project aimed at their health can improve uptake and measuring of the impact and continuity of service later. This would also give choice to a marginalised population.

Nine studies included only homeless women with children or homeless families [34, 36, 38–40, 42–45]. These studies may therefore be less representative of the typical demographics of the homeless problem-drinking population in urban settings in Europe [70] and North America [71, 72] which is largely single male. Male homeless population was included in two studies exclusively [54, 56] and five others had more than 80% men [35, 46, 47, 55, 58]. Gender differences have been seen in the homeless regarding use of health services [73, 74]. Learning the composition and preferences of the target population before setting up an intervention and tailoring the intervention to the local demographics is therefore warranted. This will ensure optimal uptake and consequently the likelihood of intervention success.

The services for homeless problem drinking people are likely varied across the globe. In the USA, the United States Interagency Council on Homelessness (USICH) is responsible for reducing homelessness [75]. It is an independent agency of the federal government designed to coordinate a response in partnership with state and local governments, and community groups. In the UK services to the homeless are provided by local councils under the guidance provided by the Department for Communities and Local Governments [76] and by charities such as Crisis, Pathway, Salvation Army, and Shelter. This means that services and their structure vary across regions. In the UK, these services are also over stretched in the face of current political and economic situation and the housing crisis since 2008, which means fewer resources are available to address malnutrition for problem drinking subgroups of homeless people.

With limited available funds, the variations in services across settings and locations can serve as an alternative to a traditional comparative study. This can identify the most beneficial interventions for a specific outcome and/or setting. Thus in future, in addition to randomized designs, natural experiments such as those designed around a change in local or regional policy, a practice within a certain hospital or a charity organisation, can provide evidence that is more applicable in terms of effectiveness. These findings should then be reported in an accessible location and format to reduce publication bias.

## Conclusions

With the high risk, single study data on any of the studied interventions we cannot conclude which intervention may be most effective or cost effective for tackling malnutrition in homeless problem-drinkers. Nevertheless, several interventions appeared able to change nutrition related behaviour in a given setting and were acceptable. Decision makers need to carefully consider which of these interventions would translate well into their own setting and population for achieving a desired outcome. Including the target population in developing intervention content and delivery may optimise intervention success.

Better quality data and long terms outcomes such as malnutrition levels, health and disease status are also needed. Comparative cost and resource use should be part of any future intervention evaluation.

## Abbreviations

ALT: Alanine transaminase
AST: Aspartate transaminase
B1: Thiamine
B12: Cobalamins
B2: Riboflavin
B3: Niacin
B5: Pantothenic acid
B6: Pyridoxine
B7: Biotin
B9: Folic acid
C: Ascorbic acid
EPOC: Cochrane Effective Practice and Organisation of Care
F: Female
G: Gram
Gp: Group
Hb: Haemoglobin
IQR: Interquartile range
Kcal: Kilocalories
kJ: Kilojoules
L: Litre
M: Male
MD: Mean difference
Mg: Milligram
Mmol: Millimoles
N: Number of participants
Nmol: Nanomoles
NR: Not reported
NV: No vitamin
P5P: Pyridoxal 5 phosphate
Pmol: Picomoles
RCT: Randomised controlled trial
SD: Standard deviation
TDP: Thiamine diphosphate
Tk: Transketolase
TPP: Thiamine pyrophosphate
UBA: Uncontrolled before and after study
V: Vitamin
Μmol: Micromoles
